# Development of tracheobronchial fluid for *in vitro* bioaccessibility assessment of particulates-bound trace elements

**DOI:** 10.1016/j.mex.2019.07.027

**Published:** 2019-08-23

**Authors:** Emmanuel Gbenga Olumayede, Ilemobayo Oguntimehin, Bolanle Babalola, Chukuwebe C. Ojiodu, Richard O. Akinyeye, Grace Olubunmi Sodipe, Joseph Uche, Ayomipo Ojo

**Affiliations:** a Department of Industrial Chemistry, Federal University, Oye, Ekiti, Nigeria; b Department of Chemical Sciences, Ondo State University of Science and Technology, Okitipupa, Ondo State, Nigeria; c Department of Animal Environment and Biology, Federal University, Oye, Ekiti, Nigeria; d Department of Science Laboratory, Yaba College of Technology, Lagos, Nigeria; e Department of Chemistry, University of Benin, Benin City, Nigeria; f Department of Industrial Chemistry, Ekiti State University, Ado-Ekiti, Nigeria

**Keywords:** In vitro bioaccessibility extraction of particulate matters-bound trace elements, Bioaccessibility, Tracheobronchial fluid, Particulate matters-bound trace elements

## Abstract

This study was piloted to evaluate bioaccessibility of particulate-bound trace elements using synthetic epithelia lung fluid; in which dipalmitoylphophatidylcholine was substituted with locus bean gum (LBSFL). The resulting data reveal that no significant change in physicochemical characteristics of the stimulated lung fluid compare with similar synthetic fluids; pH value of 7.3, density (0.998gcm^−3^), conductivity (13.9 mS m^-1^), surface viscosity (1.136 × 10^-12^ pas) and surface tension (50.6 mN m^-1^). To prove the potential applicability of the fluid in *in vitro* bioaccessibility test, we compared bioaccessibility of particulates-bound trace elements using this fluid with those of stimulated epithelial lung fluid. Bioaccessibility were relatively low values (<30%) in locus bean substituted lung fluid and stimulated epithelial lung fluid. Specifically, As and Cd had significantly higher bioaccessibility values in locus bean substituted lung fluid than stimulated epithelial lung fluid. The data demonstrate that fluid formulated and used in this study can provide a suitable means of evaluate bioaccessibility of trace elements-bound to airborne particulates.

• The fluid was used for assessing bioaccessibility of particulate matters-bound trace elements
• The formulated fluid can be applied to study in toxicity assessment
• The data can be used for inter-laboratory comparison of bioaccessibility of particulate -bound trace element and could stimulate environmental concerns on the impacts of airborne particulates.

## Graphical abstract

**Figure:**
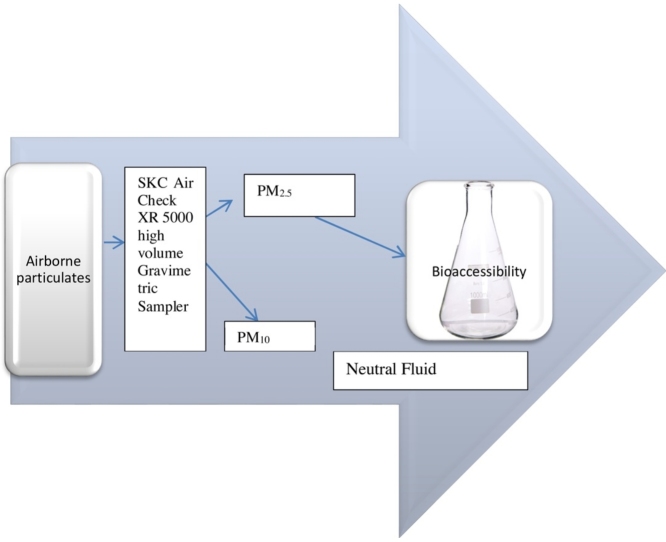

Specifications Table

**Table:** 

Subject Area:	*Environmental Science*
More specific subject area:	Bioaccessibility
Method name:	In vitro bioaccessibility extraction of particulate matters-bound trace elements
Name and reference of original method:	E.G Olumayede, I. Oguntimehin, B. Babalola, C. C. Ojiodu, R.O. Akinyeye, G. O. Sodipe, J.Uche, A. Ojo, Development of tracheobronchial fluid, *in vitro* bioaccessibility test and modeling of lung deposition of trace elements bound to airborne particulates, *Toxicology Report* (2019) (Submitted for Review)
Resource availability	Tracheobronchial fluid for *in vitro* bioaccessibility extraction of particulate matters-bound trace elements

## Method details

### Background

In risk assessment, one of the challenges for environmental toxicologist has been development of fluid with properties similar to human tracheobronchial fluids [1], so as to enable systematic investigation into bioaccessibility and lung deposition of particles in respiratory tracts. Several fluids have been explored to mimic human respiratory tract fluids in investigation of elements bioaccessibility [[2], [3], [4], [5], [6]], range from simple leaching solution to stimulated epithelial lung fluids (SELF) [2] and artificial lysosome fluid (ALF) [4], which are modification of the traditional Gamble’s solution. The use of these fluids does not represent the composition of human respiratory tract fluids which contain other substances; neutral lipids and proteins. In recent time, emerging published studies have reported crucial roles of some constituents, such as high molecular mass proteins, antioxidants and surfactants in aqueous fluids to represent the compositions of the lung fluids [7]. It is the focus of this study to develop model fluid with similar properties as tracheobronchial fluids, as this would enable a systematic investigation into the bioaccessibility and fate of deposited particles in lung to be performed.

### Materials and methods

All chemicals used in this work were of analytical grade and were used without further purification. Glycine and cysteine were purchased from Sigma Aldrich, UK. Anhydrous sodium sulphates (Na_2_SO_4_), sodium chloride (NaCl), CaCl_2_, NaHPO_4_, NaHCO_3_, KCl, MgCl_2_, were purchased from Merck, London. Potassium Chloride (KCl), were obtained from BDH. Ascorbic acid, uric acid, glutathione, albumin, cysteine, glycine and polyethylene oxide resin were products of Sigma-Aldrich. All experimental solutions were prepared using double deionized water (MilliQ system, Millipores).

### Instrumentation

Instruments used in this study included the Inductively Coupled Plasma – Mass Spectrometry (ICPMS), Sciex Elan DRC II, which was operated in standard mode equipped with a Meinhard Concentric Quartz Nebuliser, cyclonic spray chamber, and platinum skimmer and sampler cones.

#### Formulation and Preparation of locus beans substituted epithelial lung fluid

In this study, the recipe and procedures for preparation of locus bean substituted fluid was based on those of previously formulated stimulated epithelial lung fluids described in [[3], [4], [5], [6]]. Table 1 presents the compositions of stimulated epithelial lung fluids, as modified in this study, which contain Inorganic and organic constituents mixed in ultra-pure water. The modifications to the previous fluids in the present study include the use of locus beans gum, in place of dipalmitoylphophatidylcholine, a phospholipid and surface-active agent. The preparation is briefly described here,

• 1000 ml of the fluid was prepared by separate addition of the inorganic phase to organic phase.
• The inorganic phase was prepared by accurately weighed: 6020 mg NaCl, 256 mg CaCl_2_, 150 mg NaHPO_4_, 2700 mg NaHCO_3_, 298 mg KCl, 200 mg MgCl_2_, 72 mg Na_2_SO_4_ into a 500 mL HDPE and made up to the volume with ultra-pure water, and then thoroughly mixed.
• Similarly, the organic phase was prepared by accurately weighed: 18 mg ascorbic acid, 16 mg uric acid and 30 mg glutathione into a 500 mL HDPE and made up to the volume with ultra-pure water, and then thoroughly mixed.
• The two (inorganic and organic) phases were poured into 2 L conical flask containing 260 mg albumin, 122 mg cysteine, 100 mg locus bean gum, 376 mg glycine and 500 mg mucin.
• The contents were thoroughly mixed to dissolve all the components.
• The physical characteristics of the fluid; pH meter (model PHS-3C, UK), density (Pycnometer) [8], conductivity meter (Jenway, A520 model, UK), with 0.01 M of KCl as reference solution, viscosity using a capillary viscometer and For the surface tension of the fluid, the drop weight method described by [9] was adopted with slight modification.

**Table 1:** Recipe of locus bean gum substituted synthetic fluid, synthetic stimulated lung fluid and artificial lysosome fluid.

Chemicals	LBSLF (g/L)	SELF [6]	AMF (mg/100 mL) [4]
NaCl	6.020	3.21	620
Cacl_2_.2H_2_O	0.256	0.128	74
Na_2_HPO_4_	0.150	0.071	24
NaHCO_3_	2700	–	504
KCl	298	–	224
MgCl_2_	200	0.050	42
Na_2_SO_4_	72	0.039	14
DPPC	–	100	220
Locus beans gum	350	–	–
Citrate	50	20.8	–
Albumin	260		122
polyethylene oxide resins	500		
Ascorbic acid	18		
Glycine	376		
Mucin	–		1000
Glutathione			26.4

DPPC means dipalmitoylphophatidylcholine.

### Method validation

**Fig. 1:**
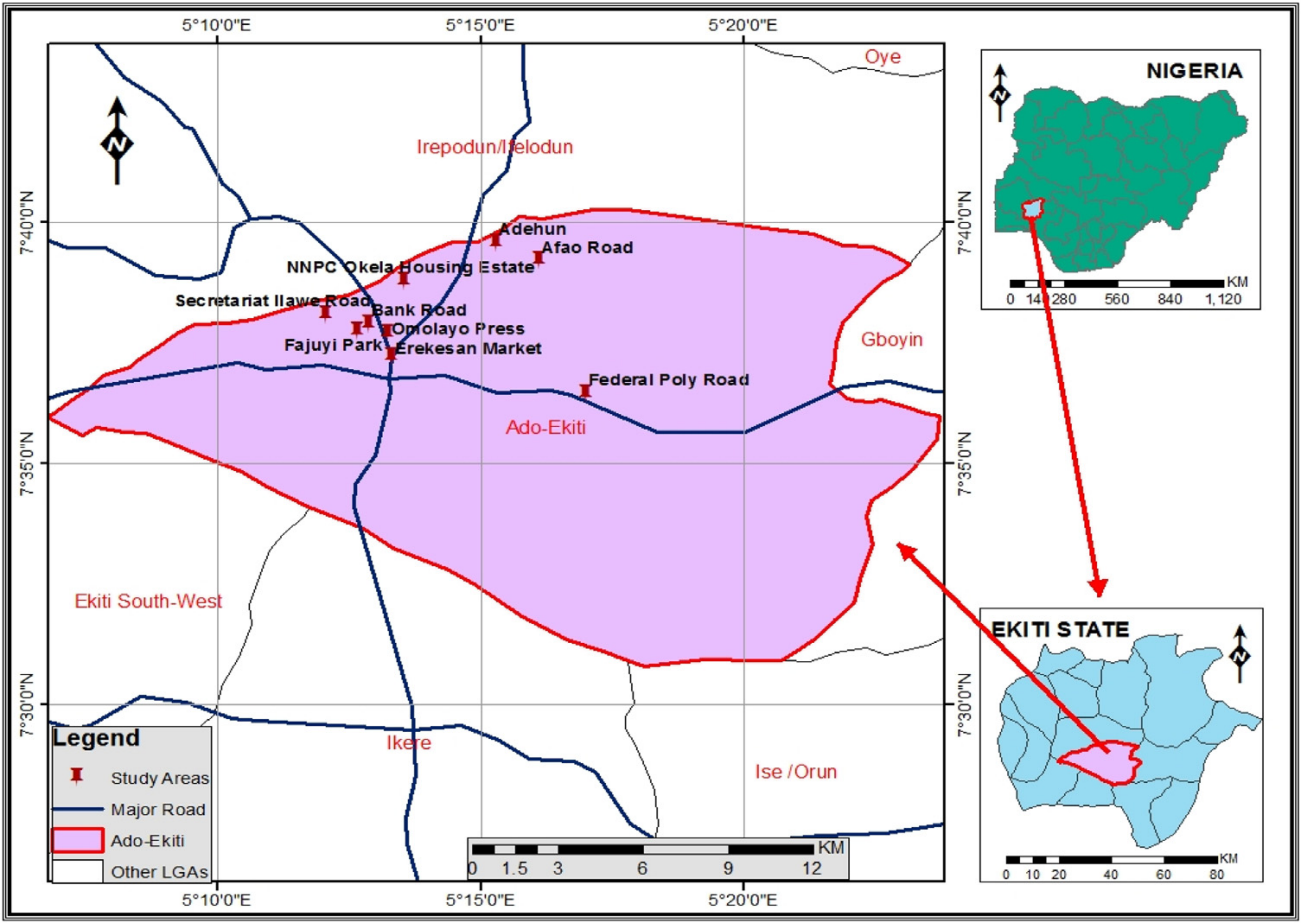
Map of Ado – Ekiti showing the coordinates of sampling sites.

The formulated LBSLF was used in *in vitro* bioaccessibility extraction of particulates-bound trace elements. For this purpose, dataset of total suspended particulate matters collected from different locations at Ado-Ekiti (Fig. 1) were used and details of sampling area, sampling methods instruments and analysis have been reported in a study [10], submitted].

#### Bioaccessibility test of elements-bound to airborne particles in the formulated fluid and other fluids

For bioaccessibility study, one quarter of the filter membranes used in samples collection was placed into three different 15 ml polypropylene centrifuge tubes, containing 10 ml each of formulated LBSLF, stimulated epithelia lung fluid (SELF) and artificial lysosome fluid (ALF). The tubes were made airtight and immersed for 2 h in a shaking water bath at 37^0^C (1-h shaking followed by 1 h still). The obtained extracts were cooled to room temperature, then centrifuged for 20 min at 3500 rpm and separated for element determination. The percentage bio accessible fraction was calculated as follows [11]:

(1) $$\text{P}\text{e}\text{r}\text{c}\text{e}\text{n}\text{t}\text{a}\text{g}\text{e}\;\text{B}\text{i}\text{o}\text{a}\text{c}\text{c}\text{e}\text{s}\text{s}\text{i}\text{b}\text{i}\text{l}\text{i}\text{t}\text{y}=\frac{\left(\text{i}\text{n}\;\text{v}\text{i}\text{t}\text{r}\text{o}\;\text{m}\text{e}\text{t}\text{a}\text{l}\right)\text{x}100}{\text{t}\text{o}\text{t}\text{a}\text{l}\;\text{m}\text{e}\text{t}\text{a}\text{l}}$$

The elemental content of the particulates were determined by Inductively Coupled plasma mass spectrometer (Aligent 5973 inert) (ICPMS). The operating conditions are: plasma flow (18.0 L/min), auxiliary flow (1.80 L/min), Nebulizer flow (1.07 L/min), sheath depth (0.30 L/min), sampling depth (5.00 min), stabilization delay (10 s), power (1.40 kW), pump rate (5 rpm).

For quality control, a standard reference material, NIST SRA 1649(Urban particulate matter) was analyzed along with blank filter. The detection limits for the methods were calculated by multiplying the standard deviation of the blank results by 3; in cases where more than one blank was used for an extraction. The concentrations of elements were calculated after subtraction of the blank filter concentration from total concentration in the particulate matters. The recovery of elements was determined by the addition of a spiking solution (prepared using deionized water and standard solution) containing 0.5 mg/L of As, Cd, Cu, Mn, Ni, Zn, Pb were conducted on LBSLF with and without sample. The percentage recovery levels were within 100 ± 15% in all the steps.

### Additional information

The importance of fluids composition on bioaccessibility of different elements has been stretched in several reports [[12], [13], [14], [15]]. The use of DPPC, a major phospholipid, in previous study does not represent the composition of native fluid, which contains other neutral lipids and proteins The major modification to these fluids in the current study is replacement of DPPC by locus bean gum. Table 2 presents the physical characteristic of the formulated LBSLF in this study, as compared with previous formulation. As shown in the table, there was no significant difference between the physical characteristic of formulated LBSLF in this study and stimulated human lung fluid reported by [6]. The pH of LBSLF in this study is 7.3, revealing the neutral nature of the fluid. The neutrality of this fluid shows that it is comparable with water and matches the pH ranges of healthy human respiratory tract (6.9–9.0) [13]. The density of fluid in the study (0.998 g cm^−3^) was almost close to 0.998gcm^−3^ previous formulation. The conductivity of 13.9 mS m^-1^ is higher than the reference solution (water) but comparable with 14.5 ± 0.1 mS m^-1^ previously reported [6]. Viscosity of 1.126pas was observed in this study compared to 1.138pas in stimulated epithelial lung fluid. Meanwhile, the surface tension of the LBSFL fluid, 50 mN m^-1^, is slightly lower than 55 mN m^-1^ reported in earlier study and 72 mN m^-1^ of reference liquid (water). The use of locust bean gum serves to improve the surface tension of formulated fluid in the present work. The observed high conductivity of the fluid compared to water has been attributed to tonicity effects of the dissolved inorganic salts [15]. Although, DPPC has been reported to influence the surface tension [16], however the use of locus bean might significantly contributed to reduce surface tension in this study.

**Table 2:** Measured physical characteristics of formulated fluid.

	pH	Density (gcm^−3^)	Conductivity (mSm^−1^)	Viscosity η” x 10^−13^ (pa s)	Surface Tension (mNm^−1^)	Reference
Stimulated lung fluid	7.2	0.999	14.5	1.138	54.9	[6]
Our formulated fluid	7.3	0.998	13.9	1.136	50.6	This study

**Fig. 2:**
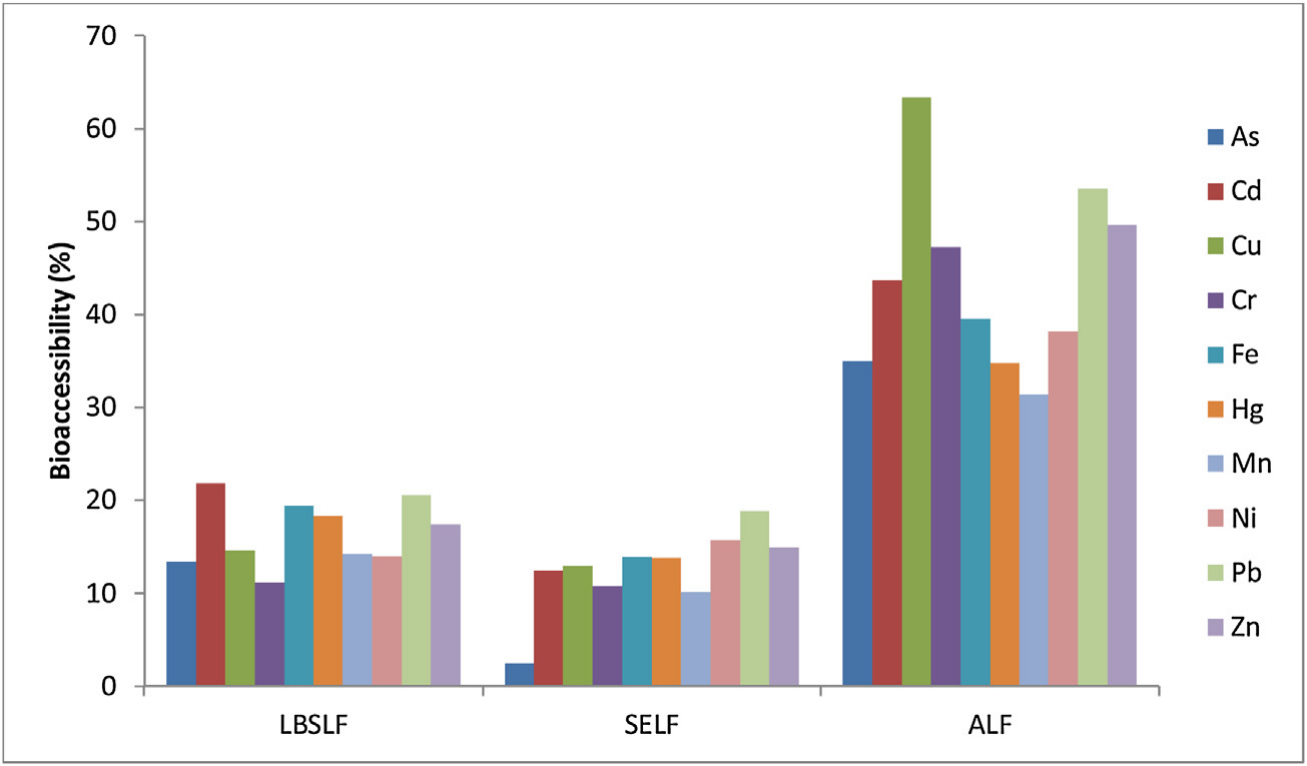
Comparison of bioaccessibility (%) of trace elements in different synthetic epithelial lung fluid.

The bioaccessibility of elements as percentage of element extractable with the formulated fluid in various sites was determined in this study. Only PM_2.5_ was considered in this study, as this is the size that can penetrate deeply to the respiratory airways. Fig. 2 presents the results of bioaccessibility assessment of elements in different fluids. The results show that bioaccessibility in the three fluids are of the order ALF > LBSLF > SELF. Bioaccessibility were relatively low values (<30%) in LBSLF and SELF. Specifically, As and Cd had significantly higher bioaccessibility values in LBSLF than SELF. Together the data demonstrate that the fluid formulated and used in this study can provide a suitable means of evaluate bioaccessibility of trace elements-bound to airborne particulates.
